# Deciphering the genetic basis of microcystin tolerance

**DOI:** 10.1186/1471-2164-15-776

**Published:** 2014-09-09

**Authors:** Anke Schwarzenberger, Thomas Sadler, Susanne Motameny, Kamel Ben-Khalifa, Peter Frommolt, Janine Altmüller, Kathryn Konrad, Eric von Elert

**Affiliations:** Cologne Biocenter, Aquatic Chemical Ecology, University of Cologne, Zuelpicher Str. 47b, 50674 Cologne, Germany; Cologne Center for Genomics, University of Cologne, Weyertal 115b, 50931 Cologne, Germany; RRZK, University of Cologne, Weyertal 121, 50931 Cologne, Germany; CECAD Cologne, University of Cologne, Robert-Koch-Str. 21, 50931 Cologne, Germany

**Keywords:** *Daphnia*, Microcystin, Tolerance, Transcriptome, Molecular basis

## Abstract

**Background:**

Cyanobacteria constitute a serious threat to freshwater ecosystems by producing toxic secondary metabolites, e.g. microcystins. These microcystins have been shown to harm livestock, pets and humans and to affect ecosystem service and functioning. Cyanobacterial blooms are increasing worldwide in intensity and frequency due to eutrophication and global warming. However, *Daphnia*, the main grazer of planktonic algae and cyanobacteria, has been shown to be able to suppress bloom-forming cyanobacteria and to adapt to cyanobacteria that produce microcystins. Since *Daphnia*’s genome was published only recently, it is now possible to elucidate the underlying molecular mechanisms of microcystin tolerance of *Daphnia.*

**Results:**

*Daphnia magna* was fed with either a cyanobacterial strain that produces microcystins or its genetically engineered microcystin knock-out mutant. Thus, it was possible to distinguish between effects due to the ingestion of cyanobacteria and effects caused specifically by microcystins. By using RNAseq the differentially expressed genes between the different treatments were analyzed and affected KOG-categories were calculated. Here we show that the expression of transporter genes in *Daphnia* was regulated as a specific response to microcystins. Subsequent qPCR and dietary supplementation with pure microcystin confirmed that the regulation of transporter gene expression was correlated with the tolerance of several *Daphnia* clones.

**Conclusions:**

Here, we were able to identify new candidate genes that specifically respond to microcystins by separating cyanobacterial effects from microcystin effects. The involvement of these candidate genes in tolerance to microcystins was validated by correlating the difference in transporter gene expression with clonal tolerance. Thus, the prevention of microcystin uptake most probably constitutes a key mechanism in the development of tolerance and adaptation of *Daphnia*. With the availability of clear candidate genes, future investigations examining the process of local adaptation of *Daphnia* populations to microcystins are now possible.

**Electronic supplementary material:**

The online version of this article (doi:10.1186/1471-2164-15-776) contains supplementary material, which is available to authorized users.

## Background

One fundamental topic in modern evolutionary ecology is the understanding of the genetic mechanisms underlying adaptation of organisms to changes in their environments. Several attempts to identify the molecular basis of adaptation have already been made for a variety of organisms: e.g. (i) Xia et al. [1] have found differences in nucleotide diversity patterns at drought-related candidate genes in two species of tomatoes indicating local adaptation. (ii) Concerning animals, Feldman et al. [2] found that adaptive evolution of the garter snake to toxic prey has occurred independently via the de novo acquisition of beneficial mutations in the skeletal muscle sodium channel. (iii) In the British peppered moth *Biston betularia*, a single core sequence has been identified to carry a signature of strong selection that is responsible for industrial melanism [3].

With the increasing availability of next-generation sequencing (NGS) technologies, it is possible to investigate the burning questions regarding mechanisms underlying adaptations also for non-model organisms with a well-understood ecology [4]. The first ecological model organism whose genome has only recently been released is *Daphnia* [5], a globally distributed grazer of algae and cyanobacteria. Its ecoresponsive genome [6, 7] and well-studied ability to adapt to many biotic and abiotic factors [8–10] makes it the perfect system with which to study evolutionary and adaptive processes on the genetic level [11].

Especially *Daphnia*’s ability to adapt to cyanobacteria and their toxins has been extensively studied during the last years [8, 10, 12]. Cyanobacteria negatively affect *Daphnia* by reducing somatic growth [13, 14] and inhibit feeding [15]. Also a decline in *Daphnia* biomass due to cyanobacteria has been observed in several field studies [16–18]. However, the generality of this negative correlation between cyanobacterial and *Daphnia* biomass has recently been questioned in an experiment [19] and in several field studies [20, 21], demonstrating that *Daphnia* have the potential to adapt to increasingly tolerate dietary cyanobacteria. Cyanobacteria and their toxins are becoming more and more of an ecological threat due to global warming and eutrophication [22], and new solutions for the management of freshwater ecosystems are needed. Therefore, it is a key issue to elucidate the underlying molecular mechanisms of *Daphnia*’s ability to tolerate cyanobacterial toxins and to therefore possibly suppress cyanobacterial blooms [19]. Toxic cyanobacterial secondary metabolites that frequently occur in cyanobacterial blooms are the well-studied microcystins and serine protease inhibitors [23, 24]. Both toxin types have been shown to negatively affect *Daphnia* [13, 14].

Identifying candidate genes is a major issue in genetics of adaptation. For cyanobacterial protease inhibitors, these candidate genes are easy to determine, as digestive proteases are the direct targets of protease inhibitors. Schwarzenberger et al. [14] found that the tolerance of different *Daphnia* genotypes depended on the residual activity of proteases; increased gene expression and enhanced activity of the non-inhibited protease type also seemed to play a role. Von Elert et al. [25] demonstrated that tolerance to cyanobacterial protease inhibitors was acquired by remodelling the affected digestive protease type.

However, in the case of microcystins, candidate genes are not as easily identified. From *in vitro* studies it has been proposed that protein phosphatases 1 and 2a are direct targets of microcystins in *Daphnia* [26]. However, it remains unclear which major physiological pathways are affected by a putative binding of microcystins to these protein phosphatases, and which of the associated elements cause the difference in tolerance among different *Daphnia* genotypes. Pflugmacher et al. [27] proposed that a glutathione-microcystin conjugate formed *in vitro* by glutathione S-transferase (GST) might be the first step in detoxification of microcystins in *Daphnia*. However, only one of twelve GST genes was found to be up-regulated in response to dietary microcystins in a recently published *D. pulex* microarray study [12], calling the role of GST as a mechanism of tolerance into question. In the same microarray study, several oxidative stress genes were up-regulated in *D. pulex* after ingestion of a microcystin producing cyanobacterium [12]. Oxidative stress responses have been observed in different aquatic organisms after exposure to microcystins [28]. However, it remains unclear whether the regulation of these genes in *Daphnia* was due to the microcystins or rather a general response to the ingestion of cyanobacteria. It is also not known whether these genes might explain tolerance to microcystins. The ingested cyanobacterium also contained other secondary metabolites; therefore the effects on gene expression could not exclusively be attributed to microcystins in this study [12]. Due to the importance of *Daphnia* as a global grazer of cyanobacteria and its capability to control cyanobacterial blooms, it is now essential to identify the candidate genes that are regulated after ingestion of microcystins. It is also essential to investigate the involvement of these candidate genes in microcystin tolerance by separating the effects of the pure microcystins from general cyanobacterial responses.

Different from Asselman [12], who only used a microcystin-producing cyanobacterium in a *Daphnia* microarray study, we here measured gene expression in transcriptomes of one tolerant clone of *D. magna*, which was fed with either 100% of a green alga or 90% of this alga with 10% of the wild-type strain of *M. aeruginosa* PCC7806 that produces both microcystins and protease inhibitors, or with 10% of its microcystin-free mutant. This cyanobacterial system, which only differed in microcystin-production, allowed disentangling effects on gene expression of *D. magna* due to microcystins from gene expression effects caused by dietary protease inhibitors. We pair-wise determined differentially expressed genes due to the different food sources and calculated significantly affected KOG-categories. From these KOG-categories we chose several candidate genes for microcystin tolerance from the comparison of green algal food/ food mixture with the wild-type strain and green alga/ mutant. By measuring differences in gene expression via qPCR in four *D. magna* clones from two ponds with or without cyanobacteria we were investigating the underlying molecular mechanisms of microcystin tolerance and local adaptation.

## Results and discussion

**Figure 1:**
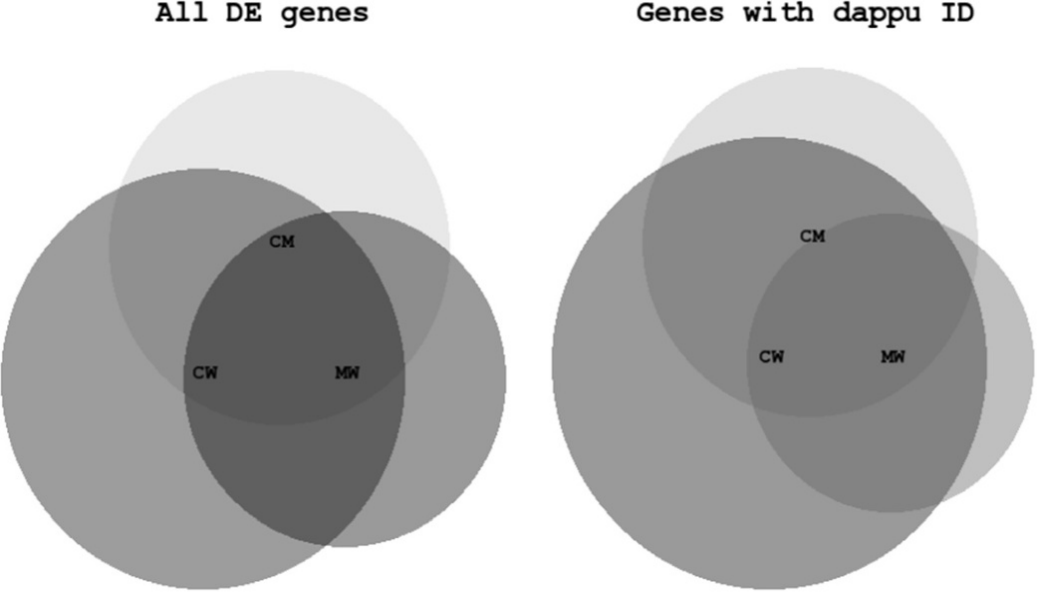
**Venn diagrams of all DE genes (CM: 1178 genes; CW: 1651; MW: 1060) and genes that could be assigned to gene IDs from the wfleabase (dappu ID; CM: 182 genes; CW: 308; MW: 130) of the three comparisons.** CW: all DE genes in the comparison between *D. magna* grown on 100% *C. klinobasis* and *D. magna* grown on 10% wild-type *M. aeruginosa* PCC7806. CM: all DE genes in the comparison between *D. magna* grown on 100% *C. klinobasis* and *D. magna* grown on 10% mutant *M. aeruginosa* PCC7806. MW: all DE genes in the comparison between *D. magna* grown on 10% wild-type *M. aeruginosa* PCC7806 and *D. magna* grown on 10% wild-type *M. aeruginosa* PCC7806. Overlapping planes share DE genes; i.e. these DE genes appear in more than one comparison. The unequal diameters of the circles in the Venn diagrams result from different numbers of DE genes.

For the transcriptome analyses we chose a clone of *Daphnia magna* (clone A [29]) that has been shown to be relatively tolerant to dietary microcystins and was therefore assumed to have distinct mechanisms for coping with dietary microcystins. In order to avoid differences in juvenile development, growth and reproduction between the cyanobacterial treatments, (which would probably obscure specific effects of microcystins on gene expression) we only added 10% of cyanobacterial carbon to the food mixtures. A significant reduction in somatic growth rate (one-way ANOVA: F_2,9_ = 53.46, p < 0.05) was observed in comparison to high quality food, while no differences in *D. magna* growth between microcystin-containing and microcystin-free cyanobacterial food was found (Tukey HSD after one-way ANOVA, p = 0.84). This indicates that *D. magna* was still able to cope with such a low concentration of microcystins without additional detectable costs besides costs associated with feeding on cyanobacteria in comparison to control food. For each pair-wise comparison between food treatments, more than 1000 differentially expressed (DE) genes were found: CM: 1178; CW: 1651; MW: 1060 (Figure 1). Due to a very strict database search of *D. magna* against the *D. pulex* genome (e-value ≤ e^-20^, score ≥ 300, similarity between sequences ≥ 40%) only around 20% of these DE genes were assigned to functional genes. This very low number probably also resulted from the fact that a high number of genes might be lineage specific for *D. magna* (comparisons with other species also resulted in less than 25% of DE genes with known functions (Additional file 1: Table S1)). Additionally, some of the DE genes might be improperly predicted by Cufflinks. However, the strictness of the database search ensured a high probability for the correct prediction of gene names and functions of the DE genes. Additionally, this strictness also enables to generalize our results for other *Daphnia* species, since these genes are probably more conserved than DE genes that were excluded. In all comparisons with WT, more DE genes were down-regulated than up-regulated (binomial tests; p < 0.05). Several serine proteases (trypsins and chymotrypsins) were regulated in response to dietary trypsin inhibitors produced both by the wild-type and the mutant of *M. aeruginosa* PCC 7806. This finding corroborates earlier findings with the same *D. magna* clone [14] obtained by qPCR.

The DE genes which could be assigned to *D. pulex* genes were assigned to KOGs and KOG-categories [30]. KOG categories, which were highly significantly affected by dietary microcystins (Table 1), contained genes which were involved in transport processes: intracellular trafficking, secretion and vesicular transport (cat. U) and secondary metabolites biosynthesis transport and catabolism (cat. Q). A total of 64 ATP-binding cassette (ABC) proteins were identified in the *D. pulex* ABC-transporter gene family, and many of them were assigned to drug efflux transporters homologous to other species [31]. It has been suggested that the increased expression of ABC transporters constitutes an adaptive mechanism enabling organisms to be resistant to drugs and toxicants [32]. Here, in *D. magna*, many transporter genes were down-regulated in response to dietary microcystins, which suggests that these transporters are involved in microcystin up-take rather than in excretion. Gene regulation of these transporter genes was probably partly responsible for the observed higher number of down-regulated in comparison to up-regulated genes in the treatments with the microcystin-producing cyanobacterium. However, a permease was up-regulated in *Daphnia*, which is in accordance with the finding that permeases have been shown to extrude cytotoxic compounds out of yeast cells [33]. Whereas transporter genes were clearly identified as novel candidate genes for microcystin tolerance, no previously suggested candidates from *in vitro* studies (i.e. glutathione synthetase and glutathione S-transferase [27]) were differentially expressed in response to microcystins. The finding that no GST gene was differentially expressed in our transcriptome is in line with Asselman et al. [12] who have found only one GST to be up-regulated. Both findings indicate a minor relevance of conjugation to glutathione for inducible tolerance to microcystins.

**Table 1:** **Gene names, accession numbers from wfleabase.org (for** ***D. pulex*** **and** ***D. magna*** **) and primer sequences used in qPCR analyses**

Gene	***D.pulex***	***D. magna***	Primer forward (5′-3′)	Primer reverse (5′-3′)
transporter of the ABC superfamily	DappuDraft_99518	m8AUGep24bs03373g21t1_ext	TCTCGGGTGGTGAAAGGAAA	ATGCCTGGACGATGTTCTGA
multidrug/pheromone exporter	DappuDraft_127589	m8AUGapi5_contig28666g180t1	CGCCATACCAATGTTCGCTT	CTGCCTGCCCAATCATTTGT
permease of the major facilitator superfamily	DappuDraft_303762	m8AUGep24bs00868g315t1_ext	CGGTTCGACTATAACGCTGC	TTCTTTGCCAGGCTTGACAC

**Figure 2:**
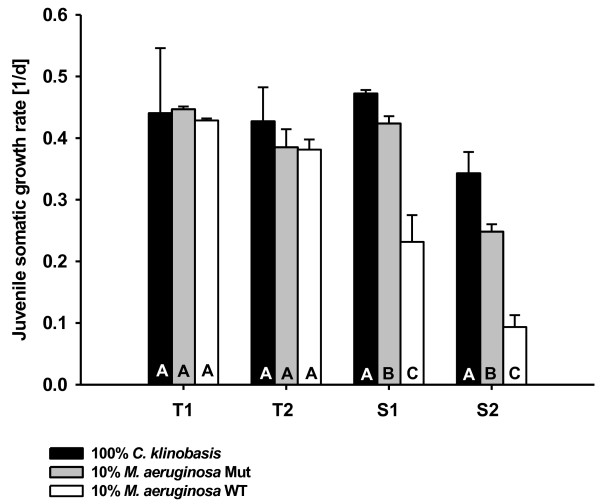
**Juvenile somatic growth rates of four clones of** ***D. magna*** **from two different populations with (T1 and T2; tolerant population) or without (S1 and S2; sensitive population) naturally occurring cyanobacteria.** *D. magna* were either grown on 100% *Chlamydomonas klinobasis* or on 90% *C. klinobasis* and 10% of either the wild-type strain of *Microcystis aeruginosa* PCC7806 or its microcystin-free mutant. Different letters indicate significant difference between treatments for each clone separately (Tukey HSD after one-way ANOVA; p < 0.05).

**Figure 3:**
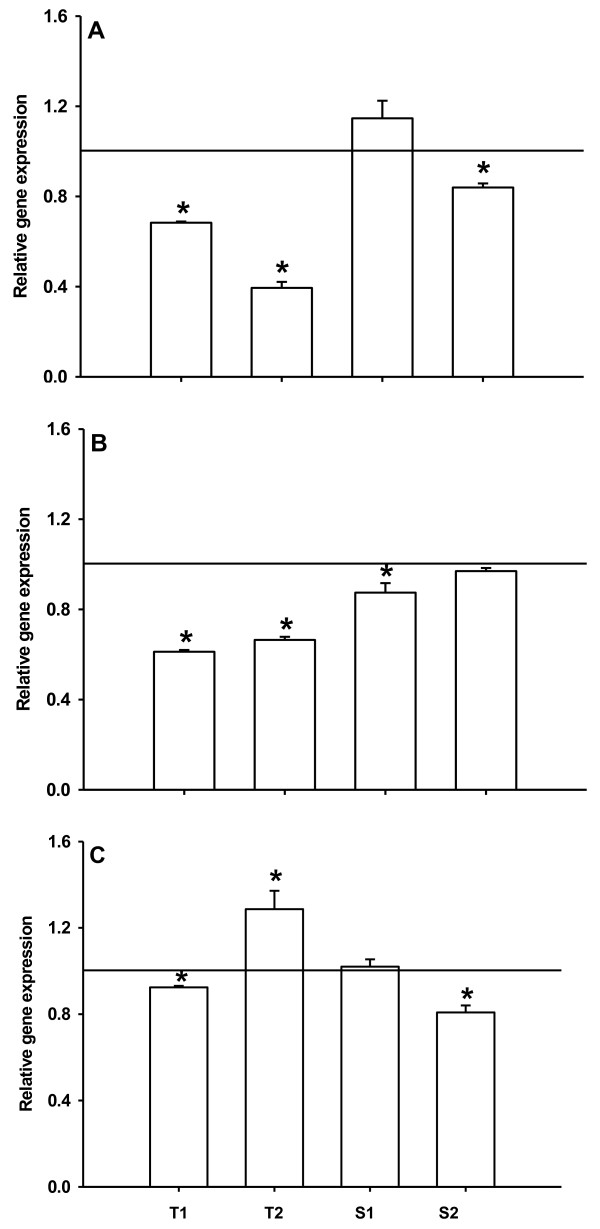
**Relative gene expression of three transporter genes (A: a transporter of the ABC superfamily, B: a multidrug/pheromone exporter, and C: a permease of the major facilitator superfamily) of four** ***D. magna*** **clones from two different populations with (T1 and T2; tolerant population) or without (S1 and S2; sensitive population) naturally occurring cyanobacteria.** *D. magna* were grown on 100% *C. klinobasis* with either control liposomes or with liposomes supplemented with microcystin LR. Asterisks indicate significant differences (p < 0.05; t-test) in gene expression in relation to gene expression on control liposomes (set to one; horizontal line).

Three differentially expressed transporter genes were chosen for candidate gene expression analyses with qPCR. These genes were a transporter of the ABC superfamily (subfamily ABCG), a multidrug/pheromone exporter (subfamily ABCB/MDR) and a permease of the major facilitator superfamily. Four *D. magna* clones were chosen for this gene expression study: Two clones showed a low tolerance to dietary microcystins (T1 and T2, Figure 2), while the other two clones had a high tolerance to the wild-type strain of *M. aeruginosa* that produces microcystins (Additional file 1: Table S1 and S2, Figure 2). In the qPCR analyses, the ABC superfamily and the multidrug/pheromone exporter genes were significantly down-regulated in three of four clones in response to supplemented pure microcystin LR (Figure 3A and B), which confirmed the results from the transcriptome study. For both genes, one of the clones with low tolerance did not regulate gene expression at all, whereas the two tolerant clones showed the strongest down-regulation of gene expression. Additionally, an expected significant increase in permease gene expression was observed only in one of the tolerant clones (Figure 3C). It is quite likely that the decrease in ABC-transporter and multidrug-transporter gene expression leads to lower production of transporter protein, which in turn results in a lower up-take of microcystins into the cells. A higher production of permeases might on the other hand cause a stronger excretion of microcystins from the cells.

Since the two tolerant clones showed a higher transporter gene regulation in response to supplemented microcystin LR than the other clones, we concluded that the regulation of transporter genes actually plays a role in tolerance to dietary microcystins. Interestingly, both tolerant clones and the clone used in the transcriptome stemmed from the same population with frequent cyanobacterial blooms [24]. Therefore, local adaptation of this population to microcystins due to higher transporter-gene regulation is plausible. Another strong indication of the involvement of transporter genes in adaptation to microcystins is a particularly high number of gene duplications observed in several ABC-transporter subfamilies [31]. For example, gene family expansions in phenotypically important genes have been shown to be responsible for adaptation of insects to insecticides [34]. Future work should therefore address the role of transporter genes in local adaptation of *Daphnia* to microcystins in natural systems, as well as how these adapted populations might be used for the management of lakes with frequent cyanobacterial blooms.

## Conclusions

Until recently it has been impossible to identify the genetic mechanisms underlying adaptive traits in non-model organisms. Whereas adaptations to natural environments have been demonstrated in ecologically relevant organisms, the genetic basis of adaptation has mainly been investigated in genetic model organisms whose adaptations to environmental variability are of minor interest. In the case of *Daphnia*, studies identifying genes that account for adaptive traits are scarce, and are based on qPCR analyses of candidate genes which were identified on the basis of *a-priori* knowledge of their function [35, 36]. By using a transcriptome-wide approach, we have been able to answer the question as to which genes are involved in microcystin tolerance. Not only did we find new candidate genes, but also demonstrated that the regulation of these genes clearly accounts for the tolerance of *Daphnia* to microcystins. In most adaptation studies, changes in DNA sequences (e.g. mutations [2], SNPs [37], etc.) were identified to constitute the genetic mechanism. Here, we suggest that in some cases adaptation of individuals or populations might arise from the adapted organism’s ability to differential express few important genes.

We here confirm that with the knowledge of the ecological background of an organism and a smart experimental design the huge amount of sequences produced in transcriptome approaches can be minimized to few genes with high potential. By releasing new ecoresponsive genomes, with *Daphnia* as a first example, and with the ever-increasing application of NGS approaches, it is now possible to link well-studied adaptive traits to the discovery of the underlying genetic mechanisms.

## Methods

### Animal cultivation and food experiment

A clone of *Daphnia magna* (clone A [29]) originating from Lake Bysjön, Sweden (where it coexisted with cyanobacteria), was cultured at 20°C in membrane-filtered tap water. Fifteen animals per litre were kept under non-limiting food concentrations (2 mg C_part_ / l) with the green alga *Chlamydomonas klinobasis* as food alga. Only third clutch neonates which had been born within 12 h were used for the experiment.

The green alga *Chlamydomonas klinobasis* was cultivated semi-continuously at 20°C in a five litre batch culture in cyanophycean medium [38] with one litre exchanged every other day. The cyanobacterium *Microcystis aeruginosa* (UTEX LB 2063 and PCC 7806) and its genetically engineered microcystin synthetase knock-out mutant (PCC 7806 mcy^─^ [39]) were cultivated in chemostat cultures (dilution rate 0.045/ d) in cyanophycean medium at 20°C. The wild-type strain PCC 7806 contains two microcystin variants (RR and LR) as well as strong trypsin inhibitors (cyanopeptolins [40, 41]). The two cyanobacterial strains only differed in the content of microcystins, while the amount of trypsin inhibitors was comparable (HPLC data not shown). All food organisms were grown under constant light (cyanobacteria: 95 μE/ m^2^/ s; *C. klinobasis* 130 μE/ m^2^/ s). Carbon concentrations (mg C/ ml) of the autotrophic food suspensions were measured for several dilutions per alga and cyanobacterium with an elemental analyser (ThermoFisher Scientific, Inc., Waltham, MA USA), and regression lines were drawn between these concentrations and the photometric light extinction at 470 nm for each dilution. These regression lines served to calculate the volume of each autotrophic food suspension needed for the carbon concentration applied in the experiments.

From a cohort of newborn *D. magna*, 15 animals each were transferred to one litre of membrane-filtered tap water and fed with 2 mg C/ l of either 100% *C. klinobasis* or 90% *C. klinobasis* and 10% of either of the two cyanobacteria. Food and medium were exchanged daily. The experiment was run in triplicate and lasted until all animals reached the size at first reproduction: Somatic growth rates of *D. magna* were determined from the dry weight of animals collected at the start and of five animals per replicate at the day when the first clutch (egg stage one) of the animals was released in the brood chamber (day seven: 100% of *C. klinobasis*; day eleven: 10% *M. aeruginosa*) as according to [42]. Levene’s tests were conducted to ensure homogeneity of variances, and growth rates were analyzed with ANOVAs and Tukey’s HSD for post-hoc comparisons. The three food treatments will be referred to as C (for 100% *C. klinobasis*), WT (for 90% *C. klinobasis* and 10% PCC 7806) and Mut (for 90% *C. klinobasis* and 10% PCC 7806 mcy-).

### Transcriptome: RNA extraction, sequencing and data analyses

At the end of the food experiment, the RNA of ten animals of each of three replicates was extracted using the RNeasy Mini Kit (Qiagen). Only animals bearing eggs at stage one [43] were chosen for RNA extraction. In order to remove any traces of genomic DNA, the RNA was treated with Desoxyribonuclease I (Fermentas) following the manufacturer’s instructions. The integrity of the RNA was verified with a 2100 bioanalyzer (Agilent). After reverse transcription and library construction, the cDNA was sequenced in paired-end mode with 36 bp read length on an Illumina Genome Analyzer IIx.

The reads of each sample were aligned to the *Daphnia magna* v. 2.4 reference assembly using TopHat version 1.2.0. By default, TopHat reports up to 40 multiple hits per read if it maps at different positions in the reference genome. Additional file 1: Table S2 provides alignment statistics for every sample. Afterwards, Cufflinks version 1.0.3 was used to identify potential transcripts and quantify their expression. Cufflinks counts reads that have multiple hits in the alignment with reduced but equal weight at each mapping position. Expression values were compared between the three groups of samples using Cuffdiff. Differentially expressed genes and isoforms were identified and FPKM values calculated. The resulting potential transcripts were blasted against the *Daphnia pulex* genome database (wfleabase.org [44]) to assign gene names and functions to the differentially expressed genes. All differentially expressed genes were classified according to eukaryotic orthologous groups [45] and KEGG metabolic pathways [46]. The number of KOGs of each of the three comparisons (either C/WT, or C/Mut, or Mut/WT) were counted and assigned to their specific category. From the whole number of KOGs from each category and the number of differentially expressed KOGs from each comparison, the significantly affected KOG categories between the comparisons were calculated with exact binomial tests (chi^2^-tests and Fisher’s exact tests) using the program Statistica 6.0. Here, we considered highly significant p-values from the chi^2^-tests to be p < 0.05, medium p-values to be p < 0.1 and low but still significant p-values to be p < 0.2.

### Liposome production

Control liposomes were produced according to [25] and subsequently enriched tenfold through centrifugation and subsequent resuspension in 1 ml liposome buffer [47]. For passive loading of the liposomes with microcystin, 500 μl of this suspension were added to 3 μg MC-LR and incubated for four h at 60°C.

### Validation of transcriptome results with qPCR

Four *D. magna* clones (T1, T2, S1 and S2) were chosen for validation of transcriptome results by qPCR. These clones were pre-cultured in the same way as clone A. In single clone experiments, five newborn *D. magna* per each of the three replicates were kept for five days on 100% *C. klinobasis* in 250 ml water. Afterwards, three replicates were fed with 100% *C. klinobasis* and 20 μl concentrated control liposomes [47] for two additional days, while three other replicates were fed with 100% *C. klinobasis* and 20 μl liposomes supplemented with microcystin LR. 20 μl of liposomes supplemented with microcystin should contain 60 ng of MC-LR. Even if 50% were lost in the experiment, this should be similar to the measured concentration used in the transcriptome study with 10% of the wild-type strain of *M. aeruginosa* PCC7806 (120 ng microcystin per litre). Food and medium were exchanged daily. After 48 h, when RNA was extracted, all animals were in the same developmental phase in all treatments.

For qPCR analyses of relative protease gene expression, RNA was extracted using the RNeasy Mini Kit (Qiagen) following the manufacturer’s instructions. RNA was purified with DNase I (Fermentas) and immediately reverse transcribed with the High-capacity cDNA Reverse Transcription Kit (ABI). The integrity of the RNA was verified with a NanoDrop. RNA concentrations were determined with a Qubit fluorometer (Invitrogen) as per the manufacturers’ instructions. QPCR and data analyses were performed as according to [48] and were close to the MIQE guidelines [49].

QPCR was conducted on a 7300 real time PCR system (Applied Biosystems). Each reaction contained 2.5 ng of cDNA template, 10 μl Power SYBR® Green PCR Master Mix (Applied Biosystems) and 2.5 μM of each primer (Table 1) in a final volume of 20 μl. Each reaction was conducted in biological triplicates. The cycling parameters were 95°C for 10 min (to activate the DNA polymerase) followed by 40 cycles at 95°C for 15 s and at 60°C for 1 min. After the actual analysis, dissociation curves were performed to verify that no primer-dimers had been amplified. For normalization, six different endogenous controls (actin, alpha-tubulin, 18S ribosomal RNA (18S), succinate dehydrogenase (SucDH), TATA-box binding protein (TBP), and ubiquitin-conjugating enzyme (UBC)) were used in qPCR analysis. These endogenous controls were chosen from a given set of ten reference genes [50].

### Availability of supporting data

NCBI Sequence Read Archive (SRA) accession numbers for the transcriptome data: Study: SRP045518; BioProject: PRJNA258118; BioSamples: SAMN02988312, SAMN02988313, SAMN02988314; Runs: SRR1552196, SRR1552197, SRR1552198, SRR1552199, SRR1552200, SRR1552201, SRR1552219, SRR1552220, SRR1552221.

## Electronic supplementary material

Additional file 1: Table S1: Database search on DE genes from the transcriptome. DE genes from the transcriptome were blasted against several databases implemented in NCBI and OrthoDB. The organisms used in these databases were either *Arthropoda*, *Crustacea* in general or a set of selected arthropod species (*Daphnia pulex*, *Drosophila melanogaster*, *Tribolium castaneum*, *Ixodes scapularis* and *Apis mellifera*). **Table S2.** TopHat Alignment Statistics. Chlamy: *D. magna* fed with 100% *Chlamydomonas klinobasis*, Mut: *D. magna* fed with 90% *C. klinobasis* and 10% of the microcystin-free mutant strain of *M. aeruginosa* PCC7806, WT: *D. magna* fed with 90% *C. klinobasis* and 10% of the WT strain of *M. aeruginosa* PCC7806. (DOCX 17 KB)
