# Supplementary material for: Deciphering the genetic basis of microcystin tolerance
Source: BMC Genomics. 2014 Sep 9;15(1):776. doi: 10.1186/1471-2164-15-776 (PMC4168211; doi:10.1186/1471-2164-15-776)
Supplement: Supplementary file 1 — Additional file 1: Table S1: Database search on DE genes from the transcriptome. DE genes from the transcriptome were blasted against several databases implemented in NCBI and OrthoDB. The organisms used in these databases were either Arthropoda, Crustacea in general or a set of selected arthropod species (Daphnia pulex, Drosophila melanogaster, Tribolium castaneum, Ixodes scapularis and Apis mellifera). Table S2. TopHat Alignment Statistics. Chlamy: D. magna fed with 100% Chlamydomonas klinobasis, Mut: D. magna fed with 90% C. klinobasis and 10% of the microcystin-free mutant strain of M. aeruginosa PCC7806, WT: D. magna fed with 90% C. klinobasis and 10% of the WT strain of M. aeruginosa PCC7806. (DOCX 17 KB) [file 12864_2014_6452_MOESM1_ESM.docx]

**Table S1**

| Database | Score ≥ 200 | similarity ≥ 40% | e-value ≤ e-20 | combined |
| --- | --- | --- | --- | --- |
| UniprotKB/swissprot (selected species) | 11% | 70% | 19% | 7% |
| UniprotKB/swissprot (*Crustacea)* | 2% | 73% | 6% | 1% |
| Refseq protein (selected species) | 24% | 64% | 37% | 21% |
| Refseq protein (*Crustacea*) | 0% | 36% | 0% | 0% |
| OrthoDB BUSCO (*Arthropoda*) | 14% | 57% | 24% | 11% |

**Database search on DE genes from the transcriptome.** DE genes from the transcriptome were blasted against several databases implemented in NCBI and OrthoDB. The organisms used in these databases were either *Arthropoda*, *Crustacea* in general or a set of selected arthropod species (*Daphnia pulex*, *Drosophila melanogaster*, *Tribolium castaneum*, *Ixodes scapularis* and *Apis mellifera*).

**Table S2**

| Sample | Total Reads | Aligned | Singletons | Multihits Pairs | Multihits  Singletons | Spliced |
| --- | --- | --- | --- | --- | --- | --- |
| ChlamyI | 6228000 | 5700046 | 403164 | 19782 | 3743 | 410356 |
| ChlamyII | 6808720 | 6150057 | 494795 | 29395 | 4753 | 447923 |
| ChlamyIII | 6888926 | 6224503 | 512171 | 25317 | 4585 | 457462 |
| MutI | 16046218 | 14547750 | 1050674 | 73593 | 64896 | 1058632 |
| MutII | 8310712 | 7523658 | 588210 | 34313 | 5153 | 556872 |
| MutIV | 7863126 | 7100179 | 581189 | 29004 | 4259 | 513200 |
| WTI | 11248752 | 10354425 | 653893 | 33845 | 4818 | 669711 |
| WTII | 10934860 | 10018107 | 648919 | 39781 | 5099 | 682411 |
| WTIII | 13483832 | 12247852 | 861428 | 56841 | 6042 | 848300 |

**TopHat Alignment Statistics.** Chlamy: *D. magna* fed with 100% *Chlamydomonas klinobasis*, Mut: *D. magna* fed with 90% *C. klinobasis* and 10% of the microcystin-free mutant strain of *M. aeruginosa* PCC7806, WT: *D. magna* fed with 90% *C. klinobasis* and 10% of the WT strain of *M. aeruginosa* PCC7806.
